# Perceived Safety Influencing Active Travel to School—A Built Environment Perspective

**DOI:** 10.3390/ijerph20021026

**Published:** 2023-01-06

**Authors:** Dorji Wangzom, Marcus White, Jeni Paay

**Affiliations:** Centre for Design Innovation, Advanced Manufacturing and Design Centre (AMDC), 469-477 Burwood Road, Hawthorn, VIC 3122, Australia

**Keywords:** active school travel, children, built-environment, traffic safety, neighborhood safety, distance

## Abstract

Despite the many research studies on active school travel (AST), the number of children walking/cycling to school is decreasing as there is a lack of implementable research evidence. This review through database searches from 2000 to 2020 aims to identify research gaps and explore new perspectives. The articles are selected and screened methodically for systematic presentation of the review. An existing active school travel framework is used to structure and discuss this review paper on mediating factors influencing children’s active travel to school, that is the perceived traffic safety, neighborhood safety, and distance to school. Perception of traffic safety could be ameliorated through lateral separation from the traffic, and this could be a new area of research. The neighborhood safety perception may require more research to validate the previous findings. Schools should be located within high-density residential development so that many children can walk to school.

## 1. Introduction

### 1.1. Background

The number of children actively commuting (i.e., walking, cycling, or other active modes) to school is decreasing in many countries around the world. The number of active commuters to school has decreased from 61% in 1991 to about 32% in 2012 in Australia [1] and active school travel (AST) is also low and decreasing in New Zealand [2]. Even in countries like Finland, Denmark, Norway, and the UK, there is a decrease in children walking or cycling independently to schools and there is an increase in car use [3]. Data from around the world show that only 40% or less than 40% of children walk to school and a small percentage cycle to school [4,5,6]. This downward trend has many implications for the health of children as the World Health Organization recommends a minimum of 60 min of physical activity in children aged 5–17 for physical, psychological, and social developmental benefits [7]. Lack of the required amount of physical activity has resulted in childhood obesity, a concern for the 21st century, and children who are obese during childhood are likely to develop diseases like diabetes and cardiovascular disease at an early age. Active travel to school is a daily physical activity in children’s lives, which could be tapped into for healthy growth and development. Recent research evidence suggests that children who actively commute to school tend to commute to other local destinations as well [8], thereby gaining the required amount of daily physical activity. Children who actively commute to school have been found to have better spatial knowledge [9], which is essential for a sense of competence and security. On the contrary, children who were non-AST were aware of destinations only and do not have an idea about the spaces between the destination and home. This shows that chauffeured children are not engaged in the space between which is crucial for learning about the environment [10]. Active travel to school is also positively associated with psychological well-being (mood or emotion) in children [11]. Urbanization has led to more vehicles on the road, access to a car, and a busy schedule for parents. The annual road traffic death is 1.35 million deaths out of which 26% represent pedestrian and cyclist deaths [12]. Among the deaths, road traffic death is highest among children and youth in the world. In the United States, traffic deaths were fewer in the early 20th century, however, they rose to the highest of 54,589 in 1972 as car ownership increased and thereafter remained relatively constant at about 40,000 per year [13]. Even in developing countries like China, the number of fatalities is on the rise. This may influence parents on how they monitor or allow their children to play in the neighborhood or walk independently to school or other local destinations.

Active school travel is influenced by several factors across different contexts, however, parental safety concerns [14], mainly the perception of traffic safety and then stranger danger has been cited as the reasons for accompanying children by car to school in countries like Denmark, Finland, the United Kingdom, and Portugal [3,15]. The parents were concerned about the stranger danger and traffic safety [8,16,17]. For others, traffic safety concerns and distance to school were identified as the main factors for active school travel [18,19,20,21,22,23].These factors have been explicitly outlined in McMillan’s active school travel framework [24] and this framework has been used to structure and discuss this review paper on different barriers to children’s active travel to school.

### 1.2. McMillan (2005) Framework

Previous research has shown that many factors influence active school travel at different levels. However, some factors are a result of the built environment, known as mediating factors in McMillan’s framework for active school travel.

The framework, specifically for active school travel from elementary school-aged children (aged 6–12) highlights that a built environment has a real/perceived impact on traffic, crime, or household transportation options. Additionally, the real/perceived impact influences parents’ decision to support active school travel. McMillan [24] suggests that built environment intervention to increase the number of children walking to school could be best achieved by focusing on the mediating factors. Factors that are a result of built environment/urban form are grouped under mediating factors and they are traffic safety (real/perceived), neighborhood safety (real/perceived), and household transportation options/distance from the school. Similarly, another set of factors known as moderating factors is outlined in the framework that do not have an apparent relationship to urban form but influences decisions on children’s active travel to school. The moderating factors are social or cultural norms, attitudes of parents, and socio-demographics. This review focuses only on perceived mediating factors as it is a result of the built environment that can be proposed for intervention.

### 1.3. Why This Review?

Despite much research on active school travel, the number of children walking/cycling to school has been decreasing. This may be because the research evidence lacks implementable results to improve active school travel. The active school travel research may require an evidence base for implementable intervention and this may be possible by adopting a future area of research to increase children walking/cycling to school. This paper identifies research gaps and proposes a way forward for a new research perspective that may be feasible to implement and may enhance active school travel. 

## 2. Methods

The database search and the selection of articles are carried out methodically for systematic presentation of the narrative review based on the paper by Green et al. [25], which provides comprehensive steps on how to conduct a narrative review. The articles are identified based on the relevance, exclusion, and inclusion criteria. The content of the paper is structured around McMillan’s [24] theoretical framework of mediating factors. Different databases are searched to gather the research evidence from 2000 to 2020 related to children’s active school travel.

The keywords/search terms used are ‘active school travel’, ‘active school travel and traffic safety perception’, ‘active school travel and stranger danger, ‘active school travel and social safety’ using databases SPORTDiscus (*n* = 5), Web of Science (*n* = 40), PubMed (*n* = 27), CINAHL (*n* = 5), ScienceDirect (*n* = 154) and Medline (*n* = 20). The papers included were from 2000–2020 and search results were screened further to include only journal research papers that had full text and were peer-reviewed. The relevant papers downloaded were further screened based on the title, abstract, and then the full text of the papers. The following were the inclusion and exclusion criteria:

Inclusion Criteria:

(i) Research for children aged 6–12 years, however, included articles that have children three years younger or older in the study group with the aforementioned age range.
(ii) Active school travel research;

Exclusion Criteria:

(i) General physical activity-related research papers;
(ii) Paper exclusively on cycling.

**Figure 1:**
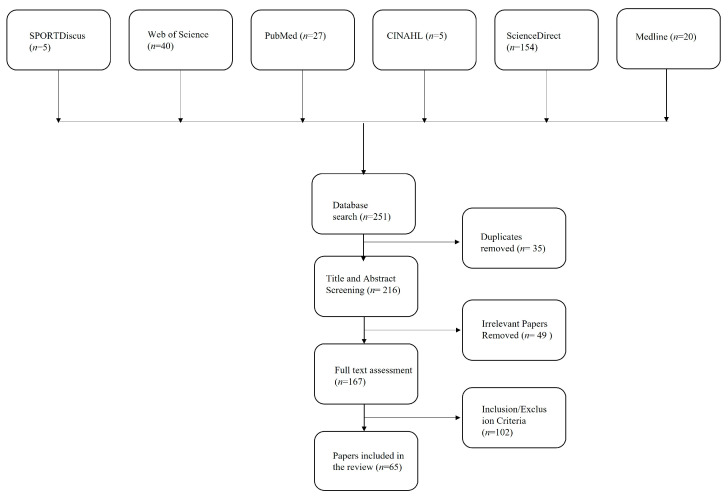
Bibliographic research flow diagram.

A total of 65 papers were included in the review of the initial 251 papers after the removal of duplicates, screening based on relevance and the inclusion/exclusion criteria as shown in the Figure 1 below. 

## 3. Results

### 3.1. Mediating Factors 

#### 3.1.1. Traffic Safety Perception

Children are the most vulnerable pedestrian group and are cognitively not well developed to navigate through the traffic environment. They are exposed to risk along the home-school route [26]. Research also shows that the majority of parents express concern for road and traffic safety [27]. This influences the decision on children’s active school travel as parents are the ultimate decision-maker for their children’s mobility [28,29]. Most of the research evidence shows a negative association between traffic safety perception and the likelihood of children’s active travel to school [16,30,31,32,33,34].Parental perception of traffic safety differs depending on the road category and the mode of active travel (i.e., walking or cycling) [35], high traffic volume/density [19,31,36,37,38], high traffic speed [37], and intersection density/dangerous intersection [36,39] that occurs along the home-school route. On the contrary, roads with low traffic speed and volume [40] and higher footpath coverage [31] were associated with less traffic risk perception. Irrespective of the traffic speed, the presence of a road with a footpath or a cycling path was perceived as safer than without these infrastructures [35]. The footpaths serve as a refuge for children from traffic. Further, a well-designed footpath without obstruction, and uneven surfaces but with curb cuts was perceived as safer from traffic dangers [41]. 

Interventions in the built environment to improve active school travel has been carried out through Safe Routes to School (US), School Travel Planning (Canada), Walking School Bus (US), Ride2School (Australia), and Safe School Travel Plans (New Zealand) [42]. As argued by McMillan [24] built environment intervention to increase active school travel may risk failure like the Safe Routes to School Program by not considering factors that mediate the relationship between active school travel and the built environment. The Safe Routes to School Program operate on direct relationship between the active school travel and the built environment/urban form. This relationship is not tested and risks failure as it does not take into account factors that mediate AST and the built environment [24]. Limited research evidence on the effectiveness of the interventions shows improvements in the rates of active school travel after the implementation of the interventions. The California Safe Route to School program showed mixed results as out of the 10 schools, only 5 schools showed a positive result on the number of children walking/biking to school, and the rest of the schools showed weak results or the same as before the intervention [43]. It proved to be less effective in increasing active school travel [44,45]. There was only a 2.1% increase in AST after the interventions were implemented [46]. Even in the Canadian School Travel Planning intervention, only 17% of the sample reported driving less to school [47]. Despite the intervention, two-thirds of the children continued to be driven to school [48]. A recent intervention in road infrastructure to improve active school travel in New Zealand resulted in traffic speed reduction but an increase in car use post-intervention [21]. In light of this, built environment interventions considering perceived factors may have a better impact on active school travel than direct built environment intervention.

Traffic speed has been identified as the main risk factor in road crashes and the severity of traffic injuries as per the World Health Organisation. Traffic speed reduction can be carried out through laws or transportation measures. However, the state laws requiring a reduction in traffic speed around schools did not increase the proportion of children walking to school nor did it result in the removal of traffic-related barriers [49]. Speed reduction can also be achieved through transportation measures and limiting the speed as in many areas of London and the UK. Measures of speed humps, chicanes, and raised junctions were placed every 100 m to establish traffic zones of 20 mph (32 km per hour). This resulted in reductions in road collisions, especially involving children [50]. However, this negatively impacts making aesthetically pleasing streetscapes as streets area also socially, functionally, and economically important.

While it will be important to provide adequate pedestrian infrastructure [51] the research quantifying the pedestrian’s perception of safety and comfort has shown that perception of traffic safety improves with lateral separation from vehicular traffic. An increase in lateral distance from vehicular traffic improves the pedestrian’s perception of traffic safety [52] and the separation elements are footpaths, footpath width, bike lanes, and buffers such as trees. This has not been explored for active school research and provides an area for future research. A comprehensive study considering different dimensions of perception would add a new perspective to this study. The active school travel research also indicated that some of these elements contribute to the perception of safety, however, may require research focusing on these aspects. Footpaths serve as a refuge from the traffic for children while walking to school and wider footpaths were perceived much safer. Additionally, parents perceived streets with trees (buffer between traffic and children) as safer for their children walking to school [41]. Street trees have been found to reduce traffic speed by creating an edge for the drivers on road, thereby increasing alertness and reducing traffic crashes [53]. There was a higher likelihood of children walking to school in an environment with a higher number of trees along the streets [54,55]. To further the research in active school travel and also to produce implementable research evidence, more research is needed on lateral separation through different separation elements such as footpath width, bike lanes, street trees, etc., and how they may enhance active school travel.

#### 3.1.2. Neighborhood Safety Perception

Neighborhood safety includes harm or abduction by a stranger (stranger danger), personal injury, and bullying [56]. Research evidence shows that parents expressed more concern for stranger danger than other dangers [57]. The more positive their perception of neighborhood safety, the higher the likelihood of children walking/cycling to school [32,58,59,60,61]. The perceived safety of the residents may not correlate to the actual crime in the area [24,56] but parents do not take the chance of bad things happening to their children [8].

Loukaitou-Sideris (2006) argued that the natural or built environment, as well as the social setting, influences the individual’s perceived risk and fear. The built environment that is not maintained well and has a run-down appearance such as broken windows [62], without proper lighting or through views, is perceived as unsafe [63]. Therefore, the built environment may have to be maintained for the perceived sense of safety, and littered or run-down areas that seem to invite crimes or unwanted activities actually decrease the sense of safety and increase crime. 

Previous research evidence on active school travel has focused on the built environment and suggests that a neighborhood with “eyes on street” design is perceived as safe by parents. The “eyes on street” design has been measured in terms of the percentage of street segments that have- more than 50% houses’ windows facing the street [32]. The “eye on the street” by the residents made children more comfortable walking to school [63]. A neighborhood that has natural surveillance [64,65], encourages pedestrians on the streets [64] and encourages interaction between neighbors [65] was perceived as safe by parents for active school travel. Foster et al. [64] argued that a walkable neighborhood (i.e., well-connected low-traffic street with access to local amenities) may encourage pedestrian presence and circulation. This is explained by Jacobs [66] as she highlights that eyes on the streets and the number of pedestrian users is interrelated. The presence of street users increases the number of eyes on the streets as nobody likes looking out to an empty street. This relationship suggests that there has to be some land uses or higher residential density along the home-school route to generate pedestrians, because as Jacobs [66] argues, a well-used street is considered safe and a deserted street unsafe. 

A neighborhood that is walkable means the amenities/land use mix are within walking distance and this will generate pedestrian traffic within the neighborhood. The association between the land use mix and active school travel has been inconsistent. Studies have shown a positive association between active school travel and higher land use mix [54,67]. On the contrary, Broberg and Sarjala [68] found a negative association between land use mix/residential density with active school travel while Rothman et al. [40] did not find any association. Su et al. [38] found that a specific land use mix of residential and institutions/government was positively associated with children’s active school travel and the presence of industrial or commercial uses along the school route may decrease the likelihood of active school travel. However, these research findings have to be interpreted cautiously as a huge area (buffer) of 100 metres is taken to calculate the land-use mix when the children interact with certain stretches of road while walking to school [69]. The neighborhood perception of safety requires more research to validate these previous findings as there is limited research evidence. Future research could focus on land uses along specific home-school routes and their association with active school travel.

### 3.2. Household Transportation Options/Distance to School

The distance to school influences the transportation option of a household [24] and it is the main factor for active school travel [18,23,70,71,72,73,74,75,76,77,78,79,80]. Research evidence has shown consistently that the shorter the distance to school, the higher the probability of children walking to school [38,58,68,81,82,83,84,85]. In the Netherlands, about 91% of the children used active travel mode to travel to school, i.e., 64% walk and 27% cycle [86], which is a high percentage of active mode users. Even in China, about 87% of the children (*n* = 765) living within the school catchment area walked to school and the average distance to school was 0.575 km [84]. In an average distance to the school of 0.76 km, about 73% of the children walked to school in Toronto, Canada [39]. Similarly, within a distance of 0.78 km, 83.5% of the total children living within this distance walked to school in Scotland [80]. Similarly, in another study, all children who walked to school lived at an average distance of 0.56 km from the school, and those who were driven lived about 3.2 km from the school [28]. 

Policies to locate schools near residential areas already exists in countries like Australia, however, the choice of school elsewhere leads to an increase in the distance feasible for active travel [72]. About 60% per cent of the primary school children in Victoria (Australia) did not attend the school closer to their home and 43% of secondary school children did not attend the nearest school [87]. Likewise, parents in New Zealand tend to select schools based on the quality and overall reputation of the school than the distance/accessibility to the school [88]. Therefore, the choice of school for the parents and children influences the distance to school, subsequently increasing car use.

The optimum distance to school that may have almost all the children walking to school varies between countries. In Dutch cities, about 70% of primary school children within a distance of 1 km from the school walked to school [58]. With increasing distances up to 5 km, there were very few children walking to school. In Japan, almost all the children enrolled in primary school walked to school and the distance to school was 3.2 km. Whereas in Australia, 35% of the children living within 0.75 km of the school were driven to school. The different thresholds for distance to school could be due to differences in context or other factors, however, shorter distances encourage more children to walk to school. The distance threshold for increased active school travel was 0.71 km (10 min walking distance) for Iran [89]. While 2 km is considered the maximum distance for walking to school in Portugal [90], the research evidence has shown that the distance for walking varies between different countries. 

Decreasing the distance to school is impracticable in already built-up areas. Besides, there are other factors in play that influence distance such as the choice or preference of a school. However, while locating new schools it could be ensured that they are in high-density residential development so that the maximum number of children can access them by walking. 

## 4. Discussion

Active school travel research has been conducted with both a broad focus on finding different association/factors [88,91] to in-depth studies of relevant factors [40,59] that influence active school travel. Over the years, much has been known about the specific factors (especially traffic safety perception and neighborhood safety perception). These studies are carried out mostly by administering survey questionnaires [16,19,27] to find the perception. Specifically, surveys with pictures of the streets, while the perception was measured by an allowance index [35] or survey with audit conducted near schools and the use of GIS to access neighborhood characteristics [30]. Research on active school travel is increasing, however it lacks implementable research evidence to enhance the number of children walking to school. This review offers new research areas for further active school travel research identified through rigorous paper searches from 6 databases from the years 2000 to 2020. While there are many factors influencing active school travel research, the mediating factors, i.e., perception of traffic safety, perception of neighborhood safety (stranger danger), and household transportation options (distance to school) are the factors that are influenced by the design of the built environment [24]. Research evidence has found that the parental perception of the neighborhood environments influences active transport and the physical activity in children [67].

Parental perception of traffic safety is negatively associated with active school travel. Interesting studies on the pedestrian level of services have shown that the perception of traffic safety can be improved through lateral separation (wider footpaths, street trees, etc.) from the traffic [52]. Additionally, there have been other studies that show that street trees decrease the speed of cars and also calm the drivers psychologically resulting in lower speeds [92]. Though studies have not been conducted for active school travel, there is some active school travel research evidence that shows that street trees improve parental perception or that wider footpaths are perceived as safer. Therefore, a new research investigation could be to find if the lateral separation (i.e., street trees, bike lanes, distance from traffic, wider footpath) from traffic has an impact on the perception of traffic safety. Future research on lateral separation from the traffic to improve the parental perception of active school travel could not only take the research in this field further but also have an impact on reducing collisions and improving the number of children walking to school, as this new research area may result in implementable built environment design solutions. 

The perception of neighborhood safety (stranger danger) is also associated with active school travel. The perception of neighborhood safety is dependent on the built environment and how it is maintained. As argued by Foster et al. [59], a walkable neighborhood provides access to amenities within walking distance, which means lower vehicular traffic volume and more pedestrians on the road. This also means parents may perceive the street to be safe from traffic for active school travel and safe from stranger danger as these streets may encourage pedestrians onto street and subsequently “eyes on the street”. Furthermore, a built environment that is maintained well and has good connectivity and views could encourage more users and a sense of safety from stranger danger. From the built environment perspective, the land use mix associated with active school travel could be an area of research. The association between active school travel and land use mix has been inconsistent and therefore a further area of research could be to take land uses specific to the school route than taking the 100 m buffer areas, used in previous research, to find the relationship. The study areas for active school travel research are defined using buffers and from within this buffer area, land use mix, density, or other built environment determinants are defined/calculated to find the association with active school travel. Research studies have considered 800 m buffers (equivalent to a 10 min walk) around the home of the child [5], while others have taken 500 m [38] or 100 m buffer widths on either side of the Global Positioning tracked trips [86]. Su et al. [38] have considered buffers of 150 m and 300 m around the child’s home to find the traffic density estimates. In practical terms, the children’s route to school covers a small area and the influence of the built environment may extend some area beyond the route. Ref. [93] have argued the unsuitability of taking a wider area when researching active school travel, as children walk to school along a road or access only a certain area or stretch, therefore, future research should focus only on actual environments used by the children while walking to school [68]. It seems that more research on neighborhood perception is necessary to validate previous research findings. 

Research evidence has consistently shown that shorter distance increases the likelihood of children walking to school. Many factors influence distance to school, for example, the choice or preference of a school. Decreasing the distance to school is impracticable in already built-up areas. However, schools should be located within high-density residential development so that many children can walk to school. While the perceived safety is important and is the focus of this review, the real safety such as crime rates may not correlate to the perceived safety and therefore the proposed interventions should focus on real as well as perceived safety. Studies have not been carried out to find specific built environment factors or factors outside the neighborhood, such as media, that have a greater influence on the travel behavior [24]. It was also found that the perception of traffic safety did not always align with the real traffic risks and there was no significant association between the perception of risk along a route and the road accidents [40]. Similarly, studies by the SWOV Institute for Road Safety Research also have found a weak positive relationship between the perceived and objective built environment determinants [94]. 

### Limitations

The identified future area of research in traffic safety perception may have significant original contribution to the field. However, the research does not take into account the busy road intersections [95,96], which also decreases the likelihood of children walking to school. A future area of research on lateral separation is around segment of road not the intersections.

## 5. Conclusions

The number of children walking or cycling to school has been decreasing in countries around the world despite increasing active school travel campaigns and research. Parents still cite traffic safety, neighborhood safety, and distance to school as the factors influencing their children’s active school travel. It appears that to take AST research further it needs to generate new knowledge that can be used to produce implementable outcomes.

The evidence indicates that the factors (perceived traffic safety, perceived neighborhood safety, household transportation options) are a result of the built environment, which could be mitigated through built environment intervention [24]. While the provision of a well-designed footpath without any obstruction is a basic requirement for an active mode of travel, interesting research [52] on the pedestrian level of services has shown that lateral distance separation from motor vehicles through the provision of buffer (increase footpath width, street trees, fences) influences the perception of traffic safety and as well as reduces traffic speed. Therefore, active school travel research on traffic safety perception’s needs to explore how lateral separation from traffic influences parental perception of traffic safety.

The neighborhood safety perceptions require more research to validate the previous findings and a possible area for future research is looking at specific land use mix along the home-school route which influences active school travel. The research evidence shows that distance to school is one of the important factors influencing active school travel, however, intervention in the built environment to reduce the distance to school may not be feasible in the already developed built environment. The location of the school within a high-density residential area may decrease the distance to the school, thereby encouraging active school travel. Shorter distances with improved perception of traffic and safety in the neighborhood should encourage active school travel.

## Data Availability

Data sharing not applicable.
